# General Therapeutics and Materia Medica, Adapted for a Medical Text-Book, with Index of Remedies, and of Diseases and Their Remedies

**Published:** 1857-12

**Authors:** 


*General Therapeutics and Materia Medica, adapted for a Medical Text-Book, with Index of Remedies, and of Diseases and their Remedies.* By Robley Dunglison, M. D., LL. D., Prof. of Medicine, etc., in the Jefferson Medical College, etc., etc. Sixth edition, revised and improved.

The numerous text-books of Prof. Dunglison have all been received with the greatest favor by the profession. The work which we have before us passed to its sixth edition; a ninth edition of Medical Dictionary has lately been issued, his New Remedies and Human Physiology, have respectively passed to the seventh and eighth editions, and his work on Practice of Medicine, though a new edition has not been issued for many years, is still an admirable guide to diagnosis and practice. These, with numerous other publications, have gained for their author a world-wide reputation as one of the most learned and accomplished men who has ever graced our ranks; and one whose assiduous labors as a teacher and writer have been most useful to practitioner and student.

The work before us needs no comment, it has been too long the standard text-book on Materia Medica in most of our colleges, and we know of *no treatise* which is so admirably adapted to the wants of the medical student, for whom it is especially intended. F.

